# USP19 deubiquitinates EWS-FLI1 to regulate Ewing sarcoma growth

**DOI:** 10.1038/s41598-018-37264-5

**Published:** 2019-01-30

**Authors:** Maria E. Gierisch, Gloria Pedot, Franziska Walser, Laura A. Lopez-Garcia, Patricia Jaaks, Felix K. Niggli, Beat W. Schäfer

**Affiliations:** Department of Oncology and Children’s Research Center, University Children´s Hospital, Steinwiesstrasse 32, 8032 Zurich, Switzerland

## Abstract

Ewing sarcoma is the second most common pediatric bone and soft tissue tumor presenting with an aggressive behavior and prevalence to metastasize. The diagnostic translocation t(22;11)(q24;12) leads to expression of the chimeric oncoprotein EWS-FLI1 which is uniquely expressed in all tumor cells and maintains their survival. Constant EWS-FLI1 protein turnover is regulated by the ubiquitin proteasome system. Here, we now identified ubiquitin specific protease 19 (USP19) as a regulator of EWS-FLI1 stability using an siRNA based screening approach. Depletion of USP19 resulted in diminished EWS-FLI1 protein levels and, vice versa, upregulation of active USP19 stabilized the fusion protein. Importantly, stabilization appears to be specific for the fusion protein as it could not be observed neither for EWSR1 nor for FLI1 wild type proteins even though USP19 binds to the N-terminal EWS region to regulate deubiquitination of both EWS-FLI1 and EWSR1. Further, stable shUSP19 depletion resulted in decreased cell growth and diminished colony forming capacity *in vitro*, and significantly delayed tumor growth *in vivo*. Our findings not only provide novel insights into the importance of the N-terminal EWSR1 domain for regulation of fusion protein stability, but also indicate that inhibition of deubiquitinating enzyme(s) might constitute a novel therapeutic strategy in treatment of Ewing sarcoma.

## Introduction

Ewing sarcoma is a rare bone and soft tissue tumor which is highly distinct from other sarcoma subtypes due to its histology, molecular characteristics and prevalence to metastasize. It most commonly arises in children and adolescents with an age dependent distribution^1,2^. Current treatment includes surgery and chemotherapy with long known standard agents like doxorubicin and vincristine^3^. High chemotherapeutic toxicity, relapsed tumors and an unfavorable prognosis of patients with metastasis are demanding for novel therapeutic strategies^4,5^.

In contrast to adult cancers harboring an accumulation of multiple driver mutations, pediatric lesions display a high frequency of chromosomal rearrangements and a comparably low mutational burden^6–8^. Indeed, recent sequencing efforts revealed that, besides a recurrent translocation, the genomic landscape of Ewing sarcoma is characterized by only very few additional mutational events^9,10^. The main genetic event therefore is the balanced translocation between chromosomes 11 and 22 leading to expression of the chimeric transcription factor EWS-FLI1, in the majority of the cases^11^. This aberrant fusion protein is indispensable for tumor formation and progression as its depletion results in tumor growth inhibition^12–14^. EWS-FLI1 induces a defined program of target gene modulation and is implicated in remodeling a wide network of protein interactions^15–20^. Given the fact that Ewing sarcoma cells are highly dependent on continuous expression of the fusion protein, reduction of EWS-FLI1 levels represents an attractive therapeutic strategy.

Regulation of protein stability occurs through a highly conserved and complex molecular machinery ensuring maintenance of cell homeostasis and response to exogenous stimuli. Most intracellular proteins are degraded by the ubiquitin proteasome system. Ubiquitin tagged proteins are either subjected to destruction or initiate signaling cascades^21,22^. The process of ubiquitin chain attachment and elongation is regulated by an enzymatic cascade whereby E3 ligases mediate the ubiquitin transfer^23^. The poly-ubiquitin chains can be removed or modified by deubiquitinating enzymes (DUBs), a family of proteins comprising around 100 members classified into six subgroups according to their catalytical core domains^24^.

Ubiquitin specific protease 19 (USP19) is a USP family member with several splice isoforms. The N-terminus is responsible for chaperone function whereas the USP domain mediates protein-protein interactions. At the C-terminal end, USP19 harbors a transmembrane domain mapping its function initially to endoplasmic-reticulum-associated degradation and cellular homeostasis by supporting protein quality control and clearing misfolded proteins, mainly in interaction with the chaperone Hsp90^25–28^. USP19 seems to contribute different biological mechanisms such as protection against muscle wasting^29–31^, formation of protein aggregation^28,32^ or cell proliferation^33,34^. There, USP19 depletion inhibited proliferation of prostate cancer and breast epithelial cell lines suggesting it to have oncogenic properties^33,34^.

We have recently identified EWS-FLI1 as a substrate of the proteasome system with a distinct turnover mediated by a single lysine acceptor site in the C-terminal FLI1 part of the fusion protein^35^. In the present study, we utilized an siRNA-based screening approach to identify the deubiquitinating enzyme USP19 as a specific modulator of EWS-FLI1 protein levels. Depletion of USP19 decreased the fusion protein and increased EWS-FLI1 ubiquitination. Most interestingly, the interaction was mediated through the N-terminal domain. USP19 inhibition subsequently resulted in decreased tumor cell growth *in vitro* and *in vivo*. Our data suggest selective EWS-FLI1 destabilization by means of DUB inhibition to be an entirely new class of targeting mechanism for the treatment of Ewing sarcoma.

## Results

### SiRNA-based screening to identify regulators of EWS-FLI1 stability

**Figure 1:**
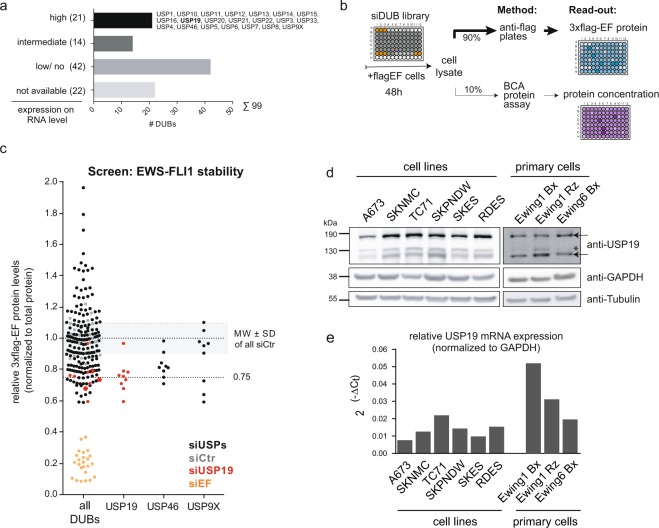
SiRNA screen identifies USP19 as a modulator of EWS-FLI1 stability. (**a**) *In-silico* selection of candidates. 21 deubiquitinating enzymes were selected based on their expression levels from publicly available microarray data sets of Ewing cell lines and tumors. (**b**) Screening setup. A673 and RDES cells stably expressing flag-tagged EWS-FLI1 were reverse transfected with single siRNAs from a small siRNA library. After 48 h, lysates were incubated in anti-flag coated plates to determine EWS-FLI1 protein normalized to total protein input. (**c**) EWS-FLI1 protein levels upon candidate knockdown. Each dot represents 3xflag-EWS-FLI1 protein levels normalized to its total protein for each single well. 3xflag-EWS-FLI1 levels upon USP19 knockdown are indicated with larger red dots and upon EWS-FLI1 knockdown in orange. (**d**) Expression levels of USP19 in indicated cell lines and primary samples were analyzed by western blot using USP19 antibody. The arrows indicate specific USP19 isoforms, asterisk marks unspecific band. (**e**) mRNA expression of USP19 was determined by quantitative RT-PCR from same cells and normalized to GAPDH.

We have previously demonstrated that EWS-FLI1 protein has a distinct turnover mediated via the ubiquitin-proteasome system with a steady-state half-life of about 2–4h^35^. As constant EWS-FLI1 protein expression is crucial for tumor cell survival^12,14^, we aimed here to decrease the half-life of the fusion protein by depleting EWS-FLI1 partner protein(s) of the ubiquitin system. Most relevantly, DUBs can rescue substrates from degradation and thereby modulate protein expression to affect regulatory pathways relevant for oncogenesis^36,37^. To identify candidate DUBs, we selected 21 enzymes of the ubiquitin-specific protease family *in silico* based on high gene expression in publicly available gene expression profiles of Ewing sarcoma cell lines and tumors (Fig. 1a, Supplementary Table ST1). Next, we established a screening strategy to directly measure steady-state EWS-FLI1 protein levels in two different cell lines (A673 and RDES) which are stably expressing a flag-tagged EWS-FLI1 at a level comparable to the endogenous protein. As read-out, we monitored the level of 3xflag-EWS-FLI1 protein in an ELISA-type assay upon transient transfection with individual siRNAs against the selected DUBs (Fig. 1b, Supplementary Table ST1). As positive control, siRNAs directed against the fusion protein were used which are downregulating both exogenous and endogenous EWS-FLI1 protein levels with similar efficiency as shown exemplarily for one siRNA in both clonal cell lines (Supplementary Fig. S1a). For the screening, all values were to total protein level per well to ensure that diminished EWS-FLI1 protein levels are not simply a result of decreased cell numbers. Using three different siRNAs for each of the 21 candidates, we identified USP19 as the main and USP46 as a second DUB as potential modulator of EWS-FLI1 protein levels. At least two siRNAs against USP19 decreased EWS-FLI1 protein levels by more than 25% in each of three screening rounds (Figs 1c and S1b) leading us to proceed with this candidate. USP9X, previously described as a DUB for the highly related E26 transformation-specific (ETS) family member ERG^38^, was also able to decrease flag-EWS-FLI1 levels albeit with only one of the three siRNA.

To validate that USP19 depletion could be relevant in Ewing sarcoma cells, we analyzed protein and mRNA expression of USP19 across six different Ewing sarcoma cell lines and three primary cell samples (Fig. 1d,e). USP19 protein presents with various isoforms of different sizes, whereby the highest band of around 150 kDa matches the size of overexpressed USP19. The amount of mRNA correlated with protein expression in all the cell lines, with TC71 displaying highest and A673 lowest levels. Hence, USP19 is indeed expressed in ES cells and could be identified as a potential novel modulator of EWS-FLI1 stability.

### USP19 specifically modulates EWS-FLI1 protein levels

**Figure 2:**
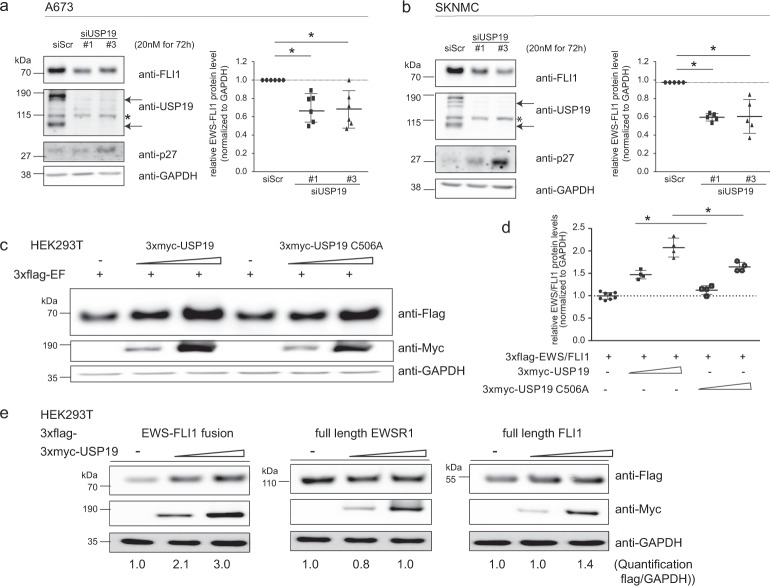
USP19 specifically modulates EWS-FLI1 protein levels. (**a**,**b**) Immunoblot analysis of USP19 depleted cells. (**a**,**b**) A673 and SKNMC cells were transiently transfected with 20 nM siRNAs for 72 h as indicated. Lysates were subjected to western blot analysis and analyzed by anti-FLI1, anti-USP19 and anti-p27 antibodies. Arrows indicate specific USP19 isoforms, asterisk marks an unspecific band. Right panel to each western blot, quantification of EWS-FLI1 proteins levels (n = 5–6, the mean is indicated by the horizontal line, error bars as SD). (**c**) Active USP19 stabilizes EWS-FLI1 protein. 3xflag-EWS-FLI1 was transiently co-expressed with a control vector or increasing levels (ratios 3xflag-EWS-FLI1 to 3xmyc-USP19 1:2 and 1:4) of wild-type or catalytically inactive 3xmyc-USP19 for 48 h in HEK293T cells. Lysates were analyzed by western blotting using anti-flag and anti-myc antibodies. (**d**) Quantification of 3xflag-EWS-FLI1 protein levels of (**c**) with n = 8 for control and n = 4 for others, the mean is indicated by the horizontal line and error bars as SD. (**e**) USP19 overexpression stabilizes specifically EWS-FLI1. 3xflag-EWS-FLI1, 3xflag-EWSR1 and 3xflag-FLI1 were transiently co-expressed with increasing concentrations (ratios 3xflag-protein to 3xmyc-USP19 1:2 and 1:4) of active 3xmyc-USP19 for 48 h in HEK293T cells. Lysates were analyzed by western blotting using anti-flag and anti-myc antibodies. Numbers below represent densitometrically quantified flag tagged protein over loading control GAPDH of a representative experiment.

To validate USP19 as a modulator of EWS-FLI1 stability, we first investigated the direct effect of USP19 depletion on endogenous EWS-FLI1 protein in two different Ewing cell lines with two different siRNAs. Similar to the initial screening, USP19 depletion resulted in reduction of USP19 protein levels and subsequent decrease of EWS-FLI1 protein by around 40% after 72 h, in both A673 and SKNMC cells (Fig. 2a,b). As control p27 protein levels also increased after depletion of the fusion protein as others have reported previously, possibly mediated by inhibition of the E3 ligase KPC1^33,34^. Transient USP19 knockdown also modulated some, but not all of the tested activated and repressed EWS-FLI1 target genes in SKNMC cells. NKX2.2, NGFR, LEMD1, LOX and ITAG11 displayed modulated expression levels, while NR0B1 or PHLDA1 were barely or not affected (Supplementary Fig. S2a). As further validation, we transiently co-expressed flag-tagged EWS-FLI1 with two increasing concentrations of 3xmyc-tagged USP19 in HEK293T cells. EWS-FLI1 levels were stabilized more than 2-fold in a dose-dependent manner by active USP19, but to a lesser extent with the catalytically inactive C506A mutant^26^, indicating that indeed the deubiquitinating activity of USP19 is involved in modulation of fusion protein levels (Fig. 2c,d). Interestingly, stabilization of EWS-FLI1 by USP19 was specific for the fusion protein as the protein levels of full length wild type EWSR1 or FLI1 remained constant upon co-expression with 3xmyc-USP19 (Fig. 2e). Our results therefore indicate that USP19 is a specific regulator of EWS-FLI1 protein stability and activity.

### USP19 interacts with the N-terminal domain of EWS-FLI1 and full length EWSR1

**Figure 3:**
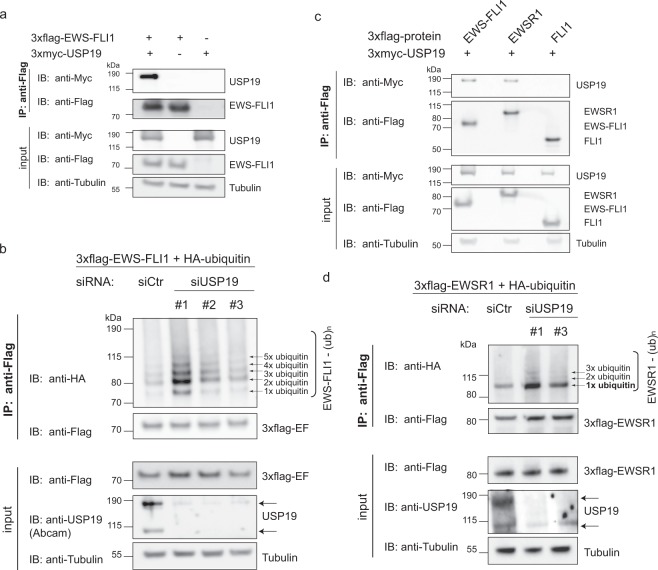
EWS-FLI1 ubiquitination is modulated by USP19. (**a**) EWS-FLI1 interacts with USP19. 3xflag-EWS-FLI1 and 3xmyc-USP19 were co-expressed in HEK293T cells for 48 h. After co-immunoprecipitation, lysates were analyzed by western blotting as indicated. (**b**) USP19 depletion increases EWS-FLI1 ubiquitination. A673 cells were transiently incubated with three different siRNAs against USP19 or a control siRNA for 24 h followed by co-expression of 3xflag-EWS-FLI1 and HA-ubiquitin for 48 h. Ubiquitination of EWS-FLI1 was analyzed by western blot analysis using anti-HA antibody. (**c**) EWSR1 also immunoprecipitates with USP19. 3xflag-EWS-FLI1, 3xflag-EWSR1 or 3xflag-FLI1 were co-expressed with 3xmyc-USP19 in HEK293 cells for 48 h. After co-immunoprecipitation, lysates were analyzed by western blotting as indicated. (**d**) USP19 depletion increases EWSR1 monoubiquitination. A673 cells were transiently incubated with two different siRNAs against USP19 or a control siRNA for 24 h followed by co-expression of 3xflag-EWSR1 and HA-ubiquitin for 48 h. Ubiquitination of EWSR1 was analyzed by western blot analysis using anti-HA antibody. Full-length blots of all immunoprecipitates are presented in Supplementary Fig. S6.

We next assessed if EWS-FLI1 and USP19 might be capable to interact. For this, tagged versions of USP19 and EWS-FLI1 were co-expressed and immunoprecipitated from HEK293T cells. We observed a consistent pull-down of USP19 with tagged EWS-FLI1 and vice versa (Figs 3a and S2b), suggesting that the two proteins interact. It was previously shown that USP19 directly deubiquitinates and rescues a variety of substrates from proteasomal degradation^26,39,40^. We therefore aimed next to investigate whether USP19 could also deubiquitinate and subsequently stabilize steady state levels of the fusion protein. We depleted USP19 with three different siRNAs prior to immunoprecipitation of tagged ubiquitinated EWS-FLI1 expressed in A673 Ewing sarcoma cells. This resulted in increased EWS-FLI1 ubiquitination compared to control treated cells (Fig. 3b). To further underscore this notion, we co-expressed 3xflag-EWS-FLI1 and HA-ubiquitin together with either active or mutant C506S 3xmyc-USP19 in A673 cells. Immunoprecipitation of tagged ubiquitinated EWS-FLI1 revealed a reduced ubiquitination pattern when co-expressed with wild type USP19 compared to co-expression with its catalytically inactive form (Supplementary Fig. S2c). Strikingly, when USP19 was co-expressed with the full length proteins EWSR1 and FLI1, only full length EWSR1 but not wild type FLI1 could be immunoprecipitated indicating that USP19 binds to the N-terminal domain of EWSR1 and EWS-FLI1 (Fig. 3c). Even though USP19 specifically modulated EWS-FLI1 but not EWSR1 protein levels (Fig. 2e), we were still interested if USP19 possibly alters EWSR1 monoubiquitination levels^35^. Indeed, depletion with  two different siRNAs of USP19 prior to immunoprecipitation of overexpressed 3xflag-EWSR1 with HA-ubiquitin suggested an increase in the monoubiquitin band, and possibly additional polyubiquitin bands of EWSR1 compared to the control (Fig. 3d).

As the main ubiquitin acceptor site for EWS-FLI1 degradation is located in the FLI1 terminus^35^, we were next interested in identifying whether USP19 still interacts with EWS-FLI1 ubiquitin acceptor site mutants. For this, we co-expressed myc-USP19 with 3xflag-EWS-FLI1 wild-type or mutants (K298R or K380R) and observed binding of USP19 to both EWS-FLI1 mutants, indicating that the interaction itself is independent of the presence of an ubiquitin chain (Fig. S2d). We further established stable reporter cell lines expressing a DsRed-IRES-EGFP-EWS-FLI1 wild-type or K380R construct (described previously^35^). The construct was lentivirally transduced (with a multiplicity of infection ~0.1) and DsRed positive SKNMC cells were sorted. Then, we depleted USP19 by siUSP19s and investigated the levels of endogenous as well as exogenous wild type or mutant EWS-FLI1 by western blotting. Most interestingly, the levels of endogenous-EWS-FLI1 and exogenous wild-type fusion protein decreased by USP19 depletion to a similar extend as observed before while the exogenous mutant form remained stable, indicating that USP19 indeed acts via the C-terminal degradation signal of the fusion protein (Supplementary Fig. S2e).

Taken together, our findings identify the N-terminal part of both EWS-FLI1 and EWSR1 as an important domain for USP19 binding which directly reduces ubiquitination levels of the fusion protein and monoubiquitination of EWSR1.

### Depletion of USP19 affects Ewing sarcoma cell growth

**Figure 4:**
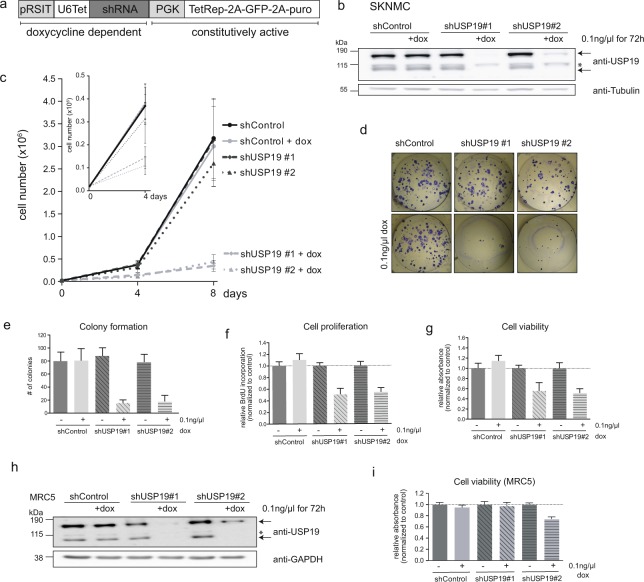
Depletion of USP19 affects Ewing sarcoma cell growth *in vitro*. (**a**) Scheme illustrating shRNA vector for stable transduction. The constitutively active part includes selection marker and a tetracycline repressive element. The doxycycline dependent part includes a tetracycline dependent promoter and the shRNA sequence. (**b**) SKNMC cells were stably transduced with two different shRNA sequences targeting USP19 and a control sequence (MOI = 5). After incubation with 0.1 ng/µl doxycycline for 72 h, USP19 protein levels were analyzed by western blotting using anti-USP19 antibody. Arrows indicate specific bands, asterisk marks unspecific band. (**c**) USP19 depletion affects cell growth. Knockdown of USP19 was induced in 2 × 10^4^ SKNMC cells as indicated and cells were counted after 4 (smaller graph) and 8 days (larger graph). Total cell numbers were plotted from three independent experiments, error bars as SD. (**d**) Depletion of USP19 affects Ewing long-term cell survival. Doxycycline induced and non-induced SKNMC cells were plated to assess colony formation after 14 days. (**e**) Quantification of colonies from (**d**) represented as total counts of colony numbers (n = 3, error bars as SD). (**f**,**g**) Knockdown of USP19 affects cell proliferation and viability. Doxycycline induced and non-induced SKNMC cells were assessed for incorporation of BrdU or incubated with WST1 reagent (**g**) or both after 96 h. Values are shown relative to untreated shControl cells (n = 3, error bars as SD). (**h**,i) USP19 depletion has limited effect in unrelated non-tumorigenic cell lines. (**h**) MRC5 cells were transduced with two different shRNA sequences targeting USP19 and a control sequence and incubated with 0.1 ng/µl doxycycline for 72 h. USP19 protein levels were assessed by western blotting using anti-USP19 antibody, arrows indicate specific bands, asterisk marks unspecific band. (i) Doxycycline induced and non-induced MRC5 cells were assessed for cell viability by WST1 after 96 h (n = 3, error bars as SD).

We next wanted to know whether DUB inhibition could serve as a possible therapeutic strategy in Ewing sarcoma. Thus we assessed whether depletion of USP19 would affect the physiology of Ewing cells. To this end, we established SKNMC and A673 cells stably expressing two inducible shUSP19 constructs which allow to deplete USP19 by addition of doxycycline. Indeed, treatment of these cells resulted in reduction of both USP19 mRNA and protein levels with the specific, but not with the control shRNA sequence (Figs 4a,b and S3a). To investigate the effect on tumor cell growth, we plated cells with and without doxycycline induction and determined cell numbers after four and eight days. Upon specific depletion of USP19, cell numbers are greatly reduced by 50% after four days and between 70–85% after eight days compared to non-induced and control shRNA cells for both Ewing cell lines (Figs 4c and S3b). Analysis of the corresponding protein levels in SKNMC cells after 72 h and eight days by western blotting revealed that depletion of USP19 is accompanied by a clear decrease in EWS-FLI1 protein levels after 8 days. This indicates that a USP19 mediated degradation of EWS-FLI1 supports the loss of Ewing sarcoma cell growth any time between 4 and 8 days (Supplementary Fig. 3c–e) where again expression levels of some, but not all of the tested target genes were modulated (Supplementary Fig. 3f). There were no significant changes in EWS-FLI1 mRNA levels (Supplementary Fig. 3f). To assess further long-term consequences, we analyzed the ability of Ewing cells to form colonies after 14 (SKNMC) and 12 days (A673), respectively. Upon USP19 depletion, colony forming capacity was reduced to less than 20% compared to non-induced and control cells which showed similar numbers of total colonies (Figs 4d,e and S3g,h). This was confirmed after 96 h where we observed further reduction in proliferation and decreased cell viability as assessed by BrdU incorporation (Fig. 4f) and WST1 assay (Fig. 4g). Conversely, transient overexpression of ectopic 3xmyc-USP19 for 48 h increased cell growth, viability and proliferation in both A673 and SKNMC cells which was not observed when a catalytically inactive mutant was expressed (Supplementary Fig. S4a–c). Interestingly, USP19 depletion in non-tumorigenic cells such as MRC5 fibroblasts or HEK293T cells did not or only slightly affect cell viability (Figs 4h,I and S3i,j). Similar growth rates of USP19-/- HEK293T cells have been described previously^25^, suggesting that USP19 supports oncogenic tumor cell growth specifically of tumor cells, despite a slight off-target effect observed for shUSP19#2. Hence, our data suggest that USP19 depletion is a relevant mechanism for Ewing cell growth inhibition *in vitro* which is mediated, at least in part, by a decrease in EWS-FLI1 protein.

### Loss of USP19 delays tumor growth *in vivo*

**Figure 5:**
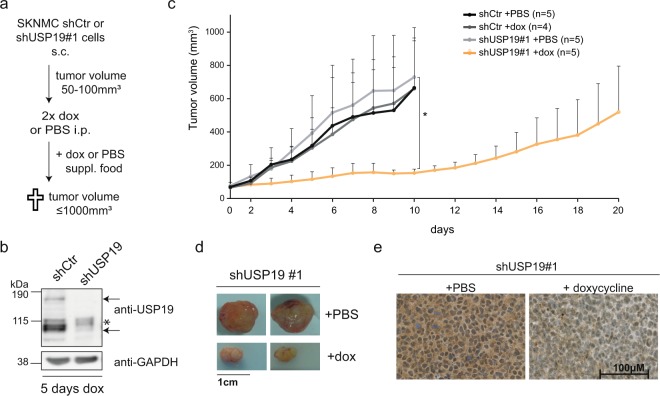
Depletion of USP19 delays tumor growth *in vivo*. (**a**) Scheme of xenograft experiments using SKNMC inducible cell lines. (**b**) Doxycycline-induced shRNA knockdown against USP19 *in vivo*. Two mice with engrafted tumors of ~200 mm^3^ (shCtr and shUSP19#1) were treated for five days with doxycycline. Tumor lysates were analyzed by western blotting with anti-USP19. (**c**) Tumor growth rate of indicated cell lines and treatment of subcutaneously injected SKNMC cells, error bars as SD. (**d**) Tumors of two representative mice transplanted with SKNMC shUSP19#1 cells and treated with doxycycline or PBS. (**e**) Immunohistochemical analysis of representative sections doxycycline or PBS treated SKNMC shUSP19#1 tumors using an USP19 antibody.

Finally, we investigated whether USP19 depletion would affect tumor growth in mouse xenografts. To this end, we subcutaneously injected inducible shControl or shUSP19#1 SKNMC cells and allowed tumors to grow up to a volume of at least 50 mm^3^ (Fig. 5a). Then, mice were intraperitoneally injected with either doxycycline or PBS for the first two days and subsequently fed with control or doxycycline supplemented food to induce and maintain USP19 knockdown. Efficient reduction of USP19 protein levels could already be seen after five days of doxycycline administration compared to control cells, as shown by both western blotting and immunohistochemistry (Figs 5b and S5a,b). While all tumors from control mice reached the final volume of 1000 mm^3^ after around ten days, tumors from doxycycline treated shUSP19 xenograft mice showed a clear delay in tumor growth. While two mice reached the maximum tumor volume only after more than double of the time period, tumors of three other mice remained stable at a volume of around 200 mm^3^ over the entire observation period (Figs 5c,d and S5c). Analysis of USP19 mRNA and protein from all tumors confirmed an efficient and selective downregulation of USP19 upon doxycycline administration, even after a longer time period (Figs 5e and S5d-e). Taken together, we could show that USP19 depletion significantly delays tumor cell growth also *in vivo* confirming our *in vitro* findings. Selective silencing of DUBs, as shown here for USP19, therefore represents a novel strategy to inhibit Ewing sarcoma tumor growth.

## Discussion

In this study, we investigated destabilization of EWS-FLI1 protein by deubiquitinating enzymes as a novel therapeutic strategy in Ewing sarcoma. In particular, we identified USP19 as a specific modulator of EWS-FLI1 protein stability which mediates an increase in fusion protein deubiquitination by binding to its N-terminal domain. The decreased EWS-FLI1 protein level upon USP19 depletion abrogated long term cell growth and delayed tumor growth in mouse xenograft experiments.

The majority of screening efforts in Ewing sarcoma utilized either single or subgroups of target genes as well as cell viability as read-outs for specificity and efficacy^41–44^. As we have recently identified EWS-FLI1 turnover as an important regulatory event in Ewing sarcoma^35^, we aimed at identifying potential stabilizers of the fusion protein, and utilize their inhibition which consequently would result in EWS-FLI1 degradation and tumor depletion. Therefore, we chose to screen for DUBs as they display a limited number of family members, harbor enzymatic activity and are currently an emerging field in drug development^36,45^. To focus on the 21 USP family members highly expressed in Ewing sarcoma cell lines and tumor samples has allowed us to perform an unbiased screen for their ability to modulate EWS-FLI1 protein levels as a read-out in two different Ewing cell lines. We chose a short 48 h incubation time to minimize secondary events due to general inhibition of cell growth. This led to the identification of USP19 as a novel regulator of EWS-FLI1 protein levels. However, it is very likely that other DUBs potentially regulate EWS-FLI1 turnover in a proteolytic or non-proteolytic manner as we could not observe more than 40% reduction in fusion protein levels upon targeting USP19 alone.

Interestingly, USP9X was identified as a DUB for ERG, an ETS family member closely related to FLI1^46^. Similar to our results, it could be demonstrated that depletion of USP9X increased ERG ubiquitination and subsequently suppressed prostate tumor growth^38^. As about 10% of Ewing sarcoma tumors carry an alternative EWS-ERG fusion, inhibition of this DUB as a therapeutic strategy might also be of interest in this subgroup of tumors. Further, USP9X regulation was also suggested to modulate wild type FLI1 stability even though only binding was shown^38^. In our screening approach, USP9X knockdown resulted in a decrease of EWS-FLI1 stability with only one siRNA which did not reach the defined threshold. However, it is possible that also USP9X might further affect EWS-FLI1 levels, maybe in combination with USP19, but this remains to be investigated.

**Figure 6:**
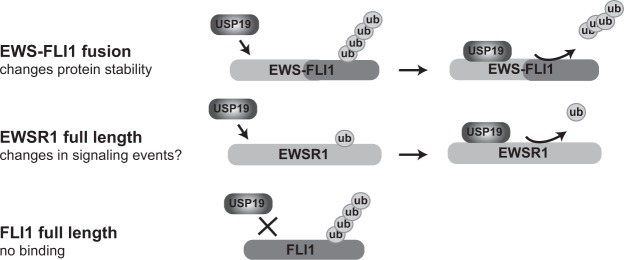
USP19 selectively stabilizes EWS-FLI1. (**a**) USP19 binds to the EWS-FLI1 fusion protein which is further deubiquitinated. (**b**) USP19 also binds to the stable EWSR1 full length protein resulting in removal of the monoubiquitin. As USP19 does not bind to full length FLI1, its polyubiquitination pattern is unaffected.

We confirmed USP19 as a stabilizer of endogenous EWS-FLI1 by analyzing steady-state fusion protein levels upon USP19 knockdown. We also observed an accompanying increase of p27 protein which has been reported previously and therefore confirms specificity of our knockdown^33,34^. In a vice versa experiment, EWS-FLI1 could be stabilized by overexpression of active USP19 in a dose dependent manner by more than 2-fold. However, also the USP19 catalytically inactive mutant was able to stabilize the fusion protein at the highest concentration indicating that a DUB-independent activity may play a role as also observed for other substrates^39,47^. Most interestingly, co-expression of active USP19 with EWS-FLI1 stabilized the fusion protein whereas full length EWSR1 and FLI1 protein levels remained unchanged even though binding of USP19 to both EWS-FLI1 and full length EWSR1 was observed in immunoprecipitation experiments. When analyzing the ubiquitination status of both proteins upon USP19 depletion, we confirmed increased ubiquitination suggesting that this is indeed the major regulatory mechanism. Hence, our data suggest the following mechanistic model (Fig. 6). USP19 binds to the EWSR1 domain of the fusion protein, as demonstrated by co-immunoprecipitation experiments, and reduces ubiquitination at an acceptor lysine site (the K380 residue^35^) in the FLI1 domain. The specificity of USP19 activity for the fusion protein therefore stems from the required presence of both domain.

USP19 was initially identified as an ER-resident DUB able to rescue degradation of ERAD substrates^26^, but has now predominantly been localized to the cytosol in association with heat shock protein 90 and other chaperones as a main substrate^27^. It is therefore likely that EWS-FLI1 interacts with USP19 in the cytosol as the fusion protein displays a constant turnover and is shuffling in and out of the nucleus^35^. Most likely, USP19, and other DUBs, modulate fusion protein levels to maintain Ewing sarcoma cell viability. However, the process by which USP19 is directed to remove EWSR1 monoubiquitination, or potential polyubiquitin chains, remains to be investigated as does the questions whether this is a general feature for other RNA-binding proteins. As this is not a signal for degradation, unlike in the case of PAX3^48^, it instead may regulate non-proteolytic activities of EWSR1, possibly at sites of DNA damage^49^.

Finally, we investigated the physiological consequences of USP19 depletion on Ewing sarcoma cell growth. For this, we used a doxycycline inducible model to deplete endogenous USP19 to ensure that no cell subpopulations were selected over time. We observed selectively diminished cell growth, proliferation and colony formation in two Ewing sarcoma cell lines upon specific USP19 depletion. However, as USP19 mediated degradation of EWS-FLI1 is observed on protein level beyond 72 h, but latest after 8 days, a larger part of the cell growth inhibition might be due to secondary effects of other USP19 substrates such as the cell cycle regulator p27, regulators of apoptosis cIAPs or the chaperone HSP90^27,33,34,39^. Even though only few USP19 substrates have been identified so far without a common ontology, USP19 expression clearly seems to be oncogenic in the context of Ewing sarcoma as USP19 depletion did not influence cell viability of control cell lines of non-tumorigenic origin. Most importantly, we also observed a significant tumor growth delay upon USP19 knockdown in an *in vivo* xenograft model. Combining USP19 inhibition with other treatment strategies such as classical chemotherapeutics, PARP inhibitors^7,50^, DNA-damaging agents^51^, inhibition of signaling pathways such as the PI3K pathway^52^, or antagonists of the Wnt pathway^53^ might further enhance its growth inhibitory properties.

Even though enhanced fusion protein degradation may not be regulated exclusively by USP19, this deubiquitinase seems to have a major impact and generally represents a novel protein class for targeting and possible combinations. In this context it is worth mentioning that fluctuations in EWS-FLI1 levels have recently been demonstrated^54^. Although cells displaying low fusion protein levels show higher invasive potential, re-establishment of higher EWS-FLI1 levels are necessary for active cell proliferation. Therefore, to keep fusion protein levels low, still appears to be attractive for Ewing sarcoma therapy since it would not affect steady-state protein levels of ubiquitously expressed full length EWSR1 or full length FLI1, an important factor in hematopoiesis, thereby potentially decreasing therapy related side-effects. In summary, our study identifies with selective targeting of EWS-FLI1 turnover a novel therapeutic approach for treatment of Ewing sarcoma.

## Material and Methods

### Cell lines

HEK293T, HEK293 and MRC5 cells were cultured in DMEM (Sigma Aldrich, Buchs, Switzerland) with 10% FBS (Sigma Aldrich), 2 mmol/L glutamine (BioConcept, Allschwil, Switzerland) and 100 U/ml penicillin/streptomycin (ThermoFisher Scientific AG, Reinach, Switzerland) at 37 °C in 5% CO_2_. All Ewing sarcoma cell lines and primary cells were cultured in RPMI medium with the same supplements. A673, RDES and primary cells were plated on 0.2% gelatin (Sigma Aldrich) pre-coated dishes. G418 selection of A673 and RDES clonal cell lines was carried out with 1.5 mg/ml G418 for one week, and kept in 0.2 mg/ml G418 for further culturing. Puromycin selection was performed with 1.5 µg/ml, whereas 0.5 µg/ml was used for maintenance. All cell lines have been tested mycoplasma negative. Ewing cells were authenticated by STR profiling in 2016/05. Primary Ewing cell lines were characterized by karyotyping at the diagnostic laboratory of the Children´s Hospital Zurich, Switzerland and described before^55^. The use of primary cells did not required ethical approval.

### Reagents and antibodies

The following reagents were used: DMSO, doxycycline (both Sigma Aldrich), G418 (Promega, Duebendorf, Switzerland), puromycin (ThermoFisher Scientific AG). The following commercial antibodies were used: anti-Flag (dilution 1:1000, clone M2, Sigma Aldrich), anti-FLI1 (1:1000, MBS300723 MyBioSource LLC, San Diego, CA, USA), anti-GAPDH (1:1000, D16H11 Cell Signaling Technology, Beverly, MA, USA), anti-HA (both 1:1000, clone 6E2 Cell Signaling Technology and 05–904 Millipore), anti-Myc (1:1000, clone 9B11 Cell Signaling Technology), anti-p27 (1:200, clone DCS-72.F6 ThermoFisher Scientific AG), anti-Tubulin (1:1000, clone DM1A Sigma Aldrich), and anti-USP19 (1:1000 (WB) A301-587A Bethyl Laboratories, Montgomery, TX, USA and 1:200 (IHC) or 1:1000 (WB) ab93159 Abcam, Cambridge, UK).

### Plasmids and cloning

The pRSIT-U6Tet-shRNA-PGK-TetRep-2A-GFP-2A-puro vector with shRNA sequences against USP19 or a negative control construct were purchased from Cellecta Inc. (Mountain View, CA, USA) with the following target sequences:

shControl: 5′-TTGGTGCTCTTCATCTTGTTG-3′.

shUSP19#1: 5′-GTGAAACAGAAGGTGCACTGC-3′.

shUSP19#2: 5′-AAGTGATGGAGACCTTGGCAC-3′.

USP19 cDNA (Addgene #36306) was introduced into pCMV-3xflag or pCDNA-3xmyc vectors (tag N-terminal of USP19) by In-Fusion cloning HD (Clontech Laboratories Inc., Mountain View, CA, USA) according to manufacturer’s protocol. USP19 C506A was mutated by site-directed mutagenesis using the forward primer 5′-CAATTAGGCAACACCGCCTTCATGAACAGCGTC-3′ and reverse primer 5′-GACGCTGTTCATGAAGGCGGTGTTGCCTAAATTG-3′. pCMV-3xflag-EWS-FLI1, pCMV-3xflag-EWSR1 and pCMV-3xflag-FLI1 have been described previously^35^. All clonings have been verified by sequencing. All experiments involving genetically modified organisms have been registered and carried out in accordance to the relevant guidelines in Switzerland.

### *In silico* candidate selection and screening setup

Twelve publicly available microarray data sets of Ewing cell lines and tumors were used to select DUB candidates (Supplementary Table ST1). Genes were ranked according to the total number of present calls from all data sets and their expression values. For the screen, cells were seeded and transfected with single siRNAs in a 96-well plate at the same time. After 48 h, cells were lysed in standard lysis buffer (50 mM Tris/HCl, 150 mM NaCl, 50 mM NaF, 5 mM Na_4_P_2_O_7_, 1 mM Na_3_VO_4_ and 10 mM ß-glycerolphosphate, 1% Triton X-100 with protease inhibitor cocktail, Complete Mini®, Sigma Aldrich) and cleared by centrifugation. For each well, 1/10 of the lysate was used to determine protein concentration by Pierce BCA Protein Assay Kit (ThermoFisher Scientific AG) and 9/10 were transferred to an anti-flag® high sensitivity M2 coated 96-well plate (Sigma Aldrich) and incubated for 2 h at room temperature while shaking. Wells were then incubated with anti-FLI1 antibody followed by HRP-linked anti-rabbit antibody, each for 2 h. To detect protein levels, wells were incubated with ready-to-use peroxidase substrate containing 3,3′,5,5′-tetramethylbenzidine (Sigma Aldrich). Absorbance was measured by ELISA reader at 640 nm. Every well was normalized to total protein.

### Transient transfection

For siRNA silencing, RNAiMAX transfection reagent was mixed with siRNAs (ThermoFisher Scientific AG) according to the manufacturer’s protocol and added to the well. Cell have been added directly on top of the mix in antibiotics-free medium (reverse transfection). For screening, three different siRNAs per gene were used (Silencer®Select, ThermoFisher Scientific AG, Supplementary Table ST2). For further experiments, the following siRNAs against USP19 have been selected: #1 (s21341) and #3 (s21339). For transient plasmid transfections, JetPrime (Polyplus Transfections, Illkirch, France) reagent was mixed with plasmids according to manufacturer’s instruction in antibiotics-free medium and added to cells for 48 h.

### Virus production and cell transduction with viral supernatant

For the production of lentiviral particles, HEK293T cells were transiently transfected with pMDL, pREV, pVSV and shRNA plasmids using JetPrime. After 24 h, medium was replaced and virus supernatant was harvested after a total of 72 h. Viral supernatant was cleared by centrifugation, filtered and concentrated (Amicon Ultra 15 mL Centrifugal Filters, Millipore). Cells were transduced with the viral supernatant supplemented with 10 µg/ml polybrene (Sigma Aldrich) by centrifugation for 1 h at 32 °C, medium was directly changed with centrifugation.

### Cell lysis and western blotting

Cells were lysed in standard lysis buffer, sonicated and cleared by centrifugation. Protein separation was performed on 4–12% BisTris NuPAGE pre-cast gels (ThermoFisher Scientific AG) and transferred to nitrocellulose membranes (GE Healthcare, Glattbrugg, Switzerland). After 1 h of blocking in 5% milk in 0.2% PBS-Tween, membranes were incubated with primary antibodies over night at 4 °C and for 2 h with HRP-linked secondary antibody at room temperature. Protein detection was carried out by chemiluminescence using ECL detection reagent (GE Healthcare) or SuperSignal^TM^ Western blotting reagent (ThermoFisher Scientific AG). Quantification of blots was performed using ImageJ (version 1.46r), Student t test *p < 0.05 if indicated, analyzed by Prism GraphPad. Full-length blots of figure blots are presented in Supplementary Fig. S6.

### Immunoprecipitations

For co-immunoprecipitation, HEK293T cells were lysed in interactor lysis buffer 1 (50 mM Tris/HCl, 150 mM NaCl, 1 mM EDTA, 0.5% Triton X-100 with protease inhibitor cocktail) and HEK293 cells in interactor lysis buffer 2 (50 mM Tris/HCl, 50 mM NaCl, 1.5 mM MgCl2, 25 mM NaF, 10 mM β-glycerolphosphate, 5 mM Na_4_P_2_O_7_, 2 mM Na_3_VO_4_, 10% glycerol 0.3% NP40 with protease inhibitor cocktail). Lysates were cleared by centrifugation and incubated with anti-Flag antibody coupled to Dynabeads ProteinG (ThermoFisher Scientific AG) for 30 min at 4 °C. After three washing steps, protein was eluted from the beads with 3xFlag-Peptide (Sigma Aldrich) at room temperature and prepared for western blot analysis.

To detect ubiquitinated proteins, cells were lysed in ubiquitin lysis buffer (2% SDS, 150 mM NaCl, 10 mM Tris/HCl, 2 mM Na_3_VO_4_, 50 mM NaF with protease inhibitor cocktail), boiled for 10 min and sonicated. Lysates were rotating for 30 min at 4 °C after dilution in nine volumes of dilution buffer (150 mM NaCl, 10 mM Tris/HCl, 2 mM EDTA, 1% Triton X-100) and cleared by centrifugation for 30 min at maximum speed. After immunoprecipitation, ubiquitinated proteins were analyzed by western blotting. Full-length blots of all immunoprecipitates are presented in Supplementary Fig. S6.

### RNA extraction and quantitative RT-PCR

Total RNA was extracted from Ewing cells using RNeasy (Qiagen, Hombrechtikon, Switzerland) according to manufacturer’s instruction. Complementary DNA synthesis was carried out using a High-Capacity Reverse Transcription Kit (ThermoFisher Scientific AG). Quantitative RT-PCR was performed using TaqMan gene expression master mix (ThermoFisher Scientific AG) and assays on demand (Applied Biosystems) with the following numbers: USP19 (Hs00324123_m1, Hs01103464_g1), EWS-FLI1 (Hs03024807_ft), GAPDH (Hs04420697_g1), HMBS (Hs00609296_g1), ITGA11 (Hs00201927_m1), LEMD1 (Hs01077215_m1), LOX (Hs00942481_m1), MAP2K6 (Hs00992389_m1), NGFR (Hs00609976_m1), NKX2.2 (Hs00159616_m1), NR0B1 (Hs00230864_m1), PHLDA1 (Hs00378285_g1). Cycle threshold (C_t_) values were normalized to GAPDH and relative expression values were calculated by the ΔΔC_t_ method^56^. Replicate values were pooled and represented as the geometric mean with a 95% confidence interval as error bars, Student t test *p < 0.05 if indicated, analyzed by Prism GraphPad.

### Immunohistochemistry

All steps including tumor fixation, embedding in paraffin and staining were carried out by Sophistolab AG (Muttenz, Switzerland).

### Functional assays

A673 and SKNMC cells were seeded in 96 well plates. For cell viability assays, cells were incubated with WST1 reagent (1:10 in medium, Sigma Aldrich) at 37 °C and absorbance was measured at 440 nm and 640 nm. For cell proliferation assays, BrdU incorporation was assessed using the Cell Proliferation ELISA Brdu chemiluminescent kit (Sigma Aldrich) according to the manufacturer’s instructions. To determine the number of attached cells, they were fixed with 4% PFA (Carl Roth) for 10 min and stained with 0.05% crystal violet solution (Sigma Aldrich) for 20 min. After a washing step in water, the dried crystal violet was dissolved in methanol and absorbance was measured at 540 nm. For colony formation, cells were seeded in a 6-well plate and stained with 0.05% crystal violet solution after 12–14 days. Colonies were counted manually.

### Xenograft studies

SKNMC cells (4 × 10^6^ cells) were engrafted subcutaneously in the left flank of NOD/Scid il2rg-/- mice (all female). Mice with a tumor volume of 50–100 mm^3^ were intraperitoneally injected with 53.3 mg/kg doxycycline or corresponding PBS for the first two days. Mice were fed with doxycycline (625 mg/kg) or PBS supplemented food (Provimi Kliba SA, Kaiseraugust, Switzerland). Tumor sizes were measured by caliper for two diameters at right angles. Tumor volume was calculated by V = 4/3*pi*((d_1_ + d_2_)/4)^3^. Termination point was reached upon a tumor volume of 1000 mm^3^. *p < 0.05. All animal experiments were performed in accordance with guidelines and regulations, were approved by the veterinary office of the Kt. Zürich and registered under the license number 206/15.

## Supplementary information

Supplementary Information

## Data Availability

Twelve publicly available microarray data sets of Ewing cell lines and tumors were used to select DUB candidates (summarized in Supplementary Table ST1). No further datasets were generated or analyzed during the current study.
