# Supplementary material for: USP19 deubiquitinates EWS-FLI1 to regulate Ewing sarcoma growth
Source: Sci Rep. 2019 Jan 30;9:951. doi: 10.1038/s41598-018-37264-5 (PMC6353870; doi:10.1038/s41598-018-37264-5)
Supplement: Supplementary file 1 — Supplementary Information [file 41598_2018_37264_MOESM1_ESM.docx]

**Supplementary information**

**USP19 deubiquitinates EWS-FLI1 to regulate Ewing sarcoma growth**

**Maria E. Gierisch#, Gloria Pedot#, Franziska Walser, Laura A. Lopez-Garcia, Patricia Jaaks, Felix K. Niggli, Beat W. Schäfer***

#contributed equally

Department of Oncology and Children’s Research Center, University Children´s Hospital, Steinwiesstrasse 32, 8032 Zurich, Switzerland

***Corresponding author:** Beat Schäfer, Department of Oncology, Children´s Hospital Zurich, Steinwiesstrasse 32, 8032 Zurich, Switzerland, beat.schaefer@kispi.uzh.ch, tel +41442667553, fax +41446348859

**Supplementary Figures and Figure Legends S1-S6**

The Supplementary Figures support data from the following main figures: S1 to Fig. 1, S2 to Fig. 2 and 3, S3 and S4 to Fig. 4, S5 to Fig. 5. Supplementary figure legends are included below each supplementary figure.

Full-length blots of main and supplementary western blot images are presented in Supplementary Fig. S6.

**Supplementary Tables ST1 and ST2**

The Supplementary Table ST1 is a summary of publicly available gene expression data sets used to select the highest expressed candidates of all deubiquitinating enzymes *in silico* which were then used to screen for their modulation of EWS-FLI1 stability.

The Supplementary Table ST2 includes the product number of the used Silencer® Select siRNA for the screening (siRNA library).

**Supplementary Figures and Figure Legends**

**Supplementary Fig. 1:**

**
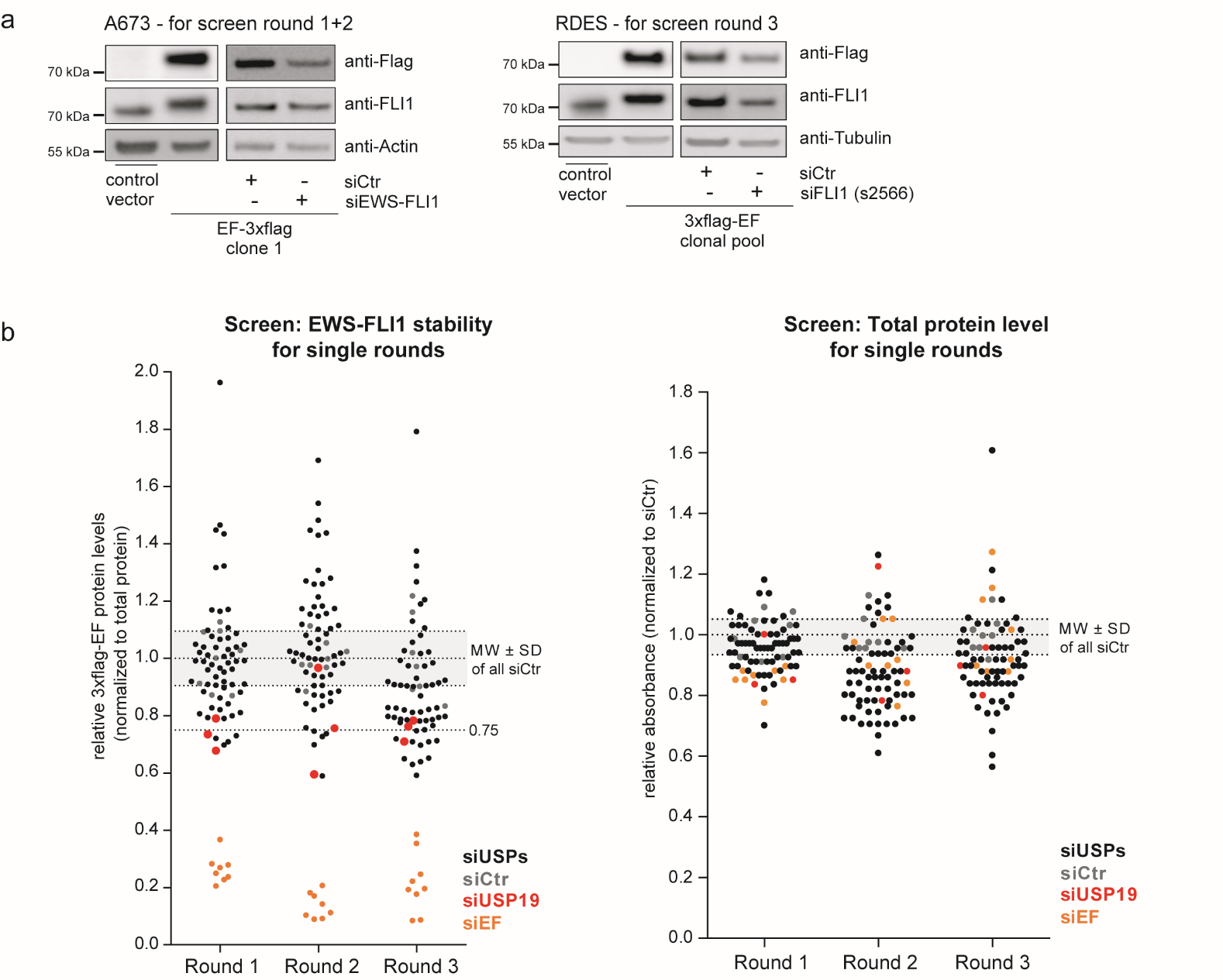
**

**Supplementary Fig. 1:** (a) Western blot analysis of lysates from A673 and RDES cell lines stably expressing 3xflag-EWS-FLI1 with anti-FLI1 and anti-Flag antibodies. Both clonal cell lines were transiently transfected with control siRNA or siRNA targeting the FLI1 part in the fusion protein. (b) Single 3xflag-EWS-FLI1 levels from the three rounds of screening (left) and total protein levels (right) upon candidate knockdown. Each dot represents a single well used to calculate ratios for Fig. 1c. Protein levels upon USP19 knockdown are indicated with red dots and for EWS-FLI1 knockdown in orange.

**Supplementary Fig. 2:**

**
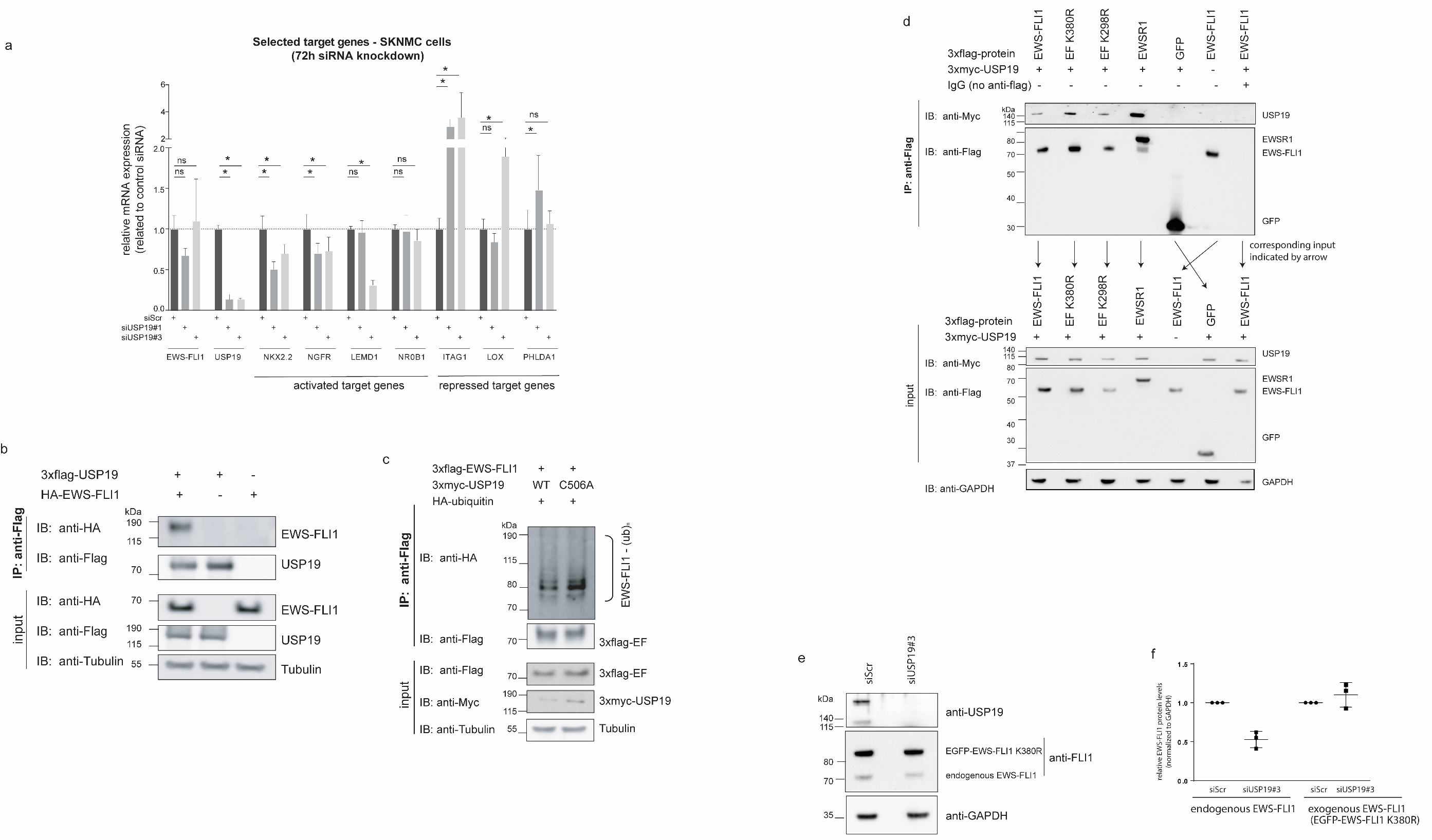
**

**
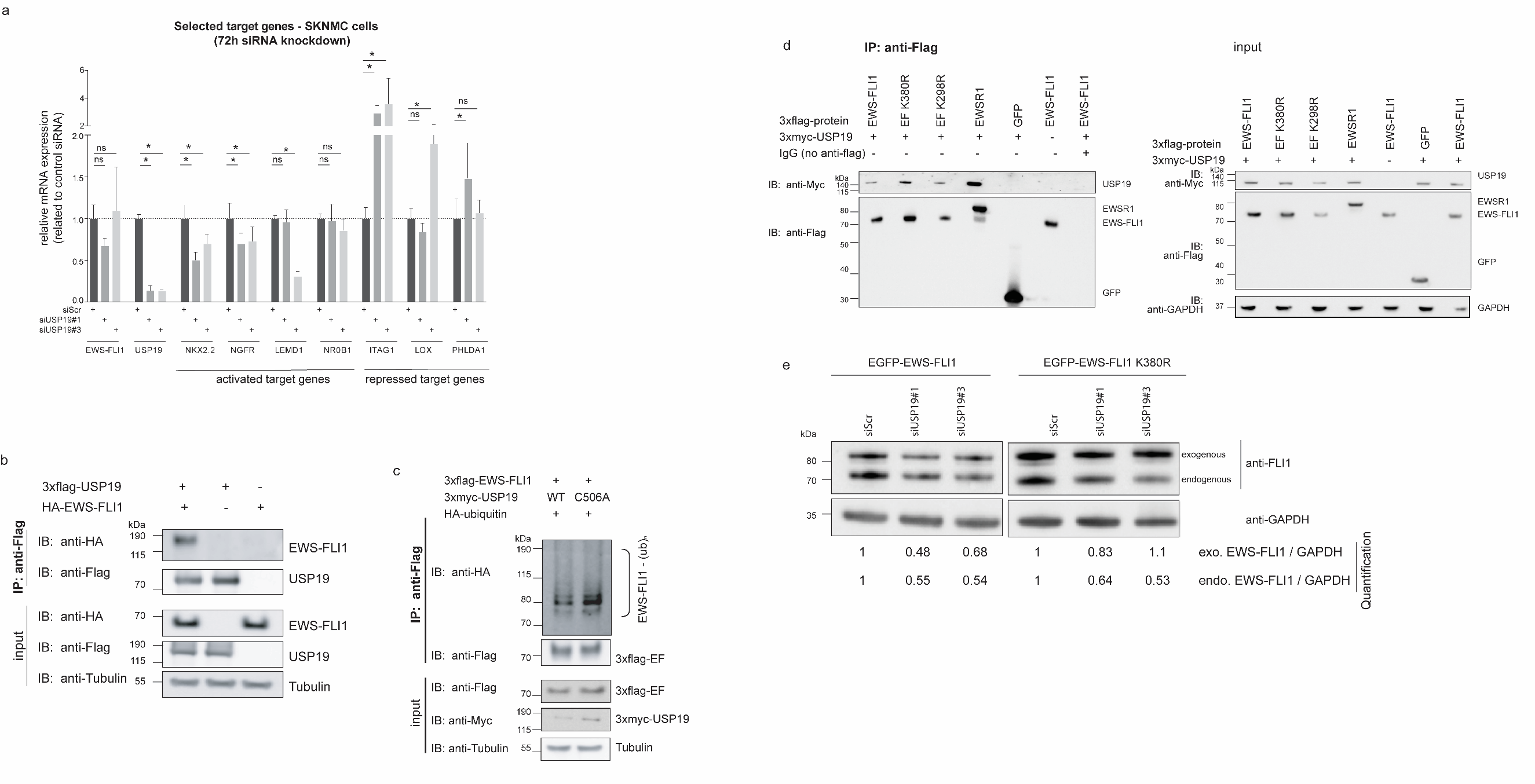
**

**Supplementary Fig. 2:** (a) USP19 knockdown modulates a subset of EWS-FLI1 target genes. SKNMC cells were transiently transfected with 20nM siRNAs for 72h as indicated and expression of indicated target genes was analyzed by quantitative RT-PCR and related to control siRNA for each gene (n=6, geometric mean, error bars as 95% confidence interval), ns = not significant, *p<0.05. (b) USP19 interacts with EWS-FLI1. 3xflag-USP19 and HA-EWS-FLI1 were co-expressed in HEK293T cells for 48h. After co-immunoprecipitation, lysates were analyzed by western blotting as indicated. (c) EWS-FLI1 ubiquitination is decreased upon active USP19 expression. 3xflag-EWS-FLI1 and HA-ubiquitin were co-expressed with either wild type or a catalytically inactive USP19 for 48h in A673 cells. After immunoprecipitation of 3xflag-EWS-FLI1, ubiquitination was determined by anti-HA antibody. (d) USP19 interacts also with EWS-FLI1 ubiquitin acceptor mutants K298R and K380R. 3xflag-EWS-FLI1, single mutants and 3xflag-EWSR1 were co-expressed with 3xmyc-USP19 in HEK293 cells for 48h. After co-immunoprecipitation, lysates were analyzed by western blotting as indicated. For the last sample, IgG was used instead of anti-flag antibody for pulldown as an additional control. (e) USP19 depletion decreases endogenous and exogenous wild type, but not ubiquitin acceptor mutant EWS-FLI1. SKNMC reporter cell lines (DsRed-IRES-EGFP-EWS-FLI1 wild type or K380R) were transiently transfected with 20nM siRNAs for 72h (as indicated and as in Fig. 2a). Lysates were subjected to western blot analysis and analyzed by anti-FLI1 antibody. Numbers below represent separately densitometrically quantified endogenous or exogenous EWS-FLI1 over loading control GAPDH. Full-length blots of immunoprecipitates are presented in Supplementary Fig. S6.

**Supplementary Fig. 3:**

**
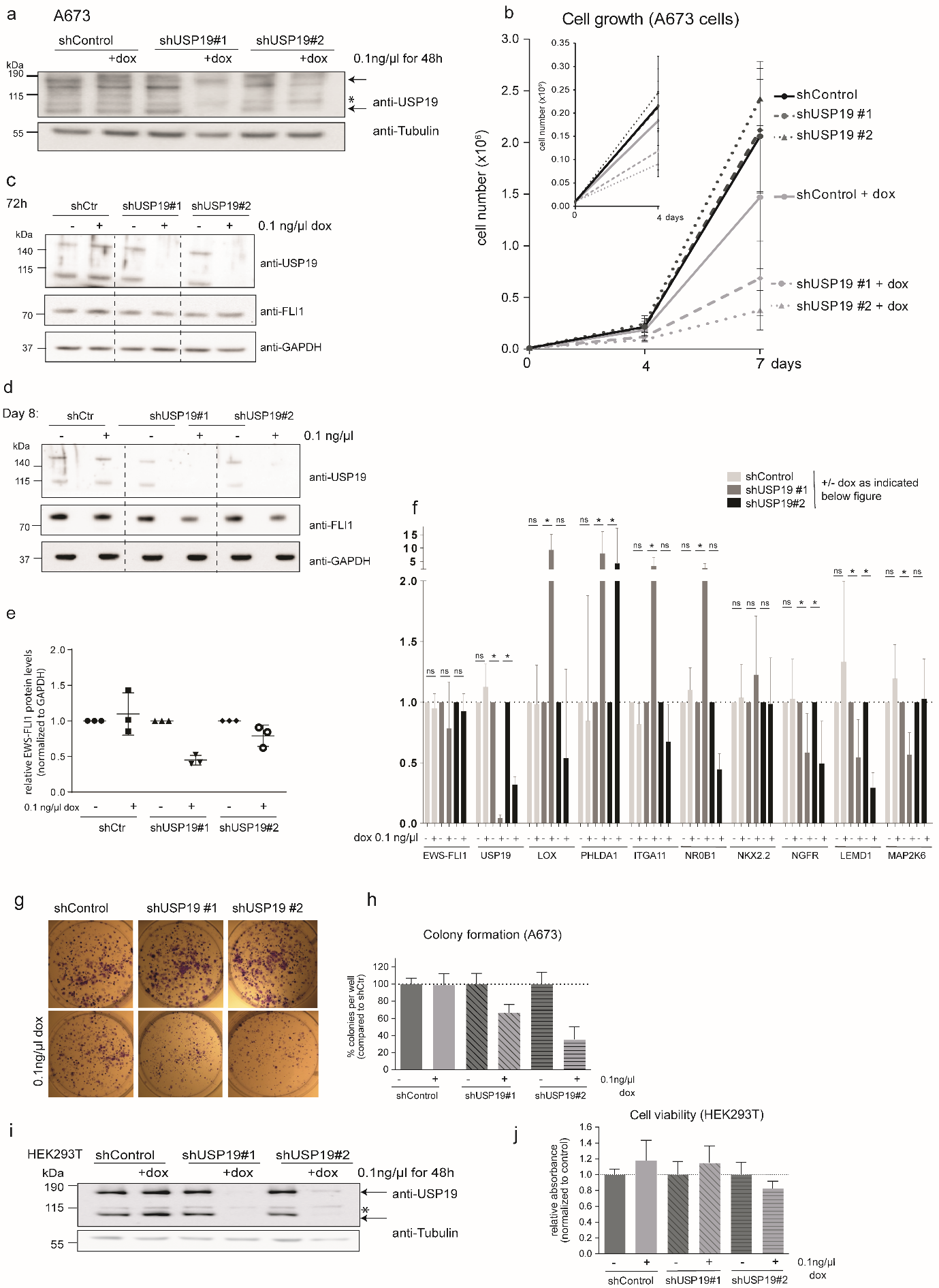
**

**Supplementary Fig. 3:** (a) A673 cells were stably transduced with two different shRNA sequences targeting USP19 and a control sequence. After incubation with 0.1ng/µl doxycycline for 72h, USP19 protein levels were analyzed by western blotting using anti-USP19 antibody, arrows indicate specific bands, asterix marks unspecific band. (b) USP19 depletion affects cell growth. Knockdown of USP19 was induced in 1x10^4^ A673 cells as indicated and cells were counted after 4 (smaller graph) and 7 days (larger graph). Total cell numbers were plotted from three independent experiments, error bars as SD. (c-f) Molecular effects of USP19 depletion for 4 and 8 days in SKNMC cells from Fig 4c. (c-d) 3 and 8 day lysates from one representative experiment were subjected to western blotting and analyzed by anti-FLI1 and anti-USP19 antibodies. (e) Quantification of EWS-FLI1 protein levels of (d) with n=3, error bars as SD. (f) Target gene pattern upon shUSP19 depletion. 8 day lysates were subjected for qRT-PCR. Delta-CT numbers were normalized to GAPDH and represented as induced over non-induced ratios for individual each target gene, n=3-6, error bars as SD. (g-h) Depletion of USP19 affects Ewing cell long-term cell survival. Doxycyline induced and non-induced A673 cells were assessed colony formation after 12 days. Values are represented as induced over not-induced ratios for each cell line (n=3, error bars as SD). (i-j) USP19 depletion has limited to no effect in unrelated non-tumorigenic cell lines. (i) HEK293T cells were transduced with two different shRNA sequences targeting USP19 and a control sequence and incubated with 0.1ng/µl doxycycline for 72h. USP19 protein levels were assessed by western blotting using anti-USP19 antibody, arrows indicate specific bands, asterix marks unspecific band. Doxycyline induced and non-induced (i) HEK293T cells were assessed for cell viability by WST1 after 96h (two independent experiments with n=6 biological replicates each).

**Supplementary Fig. 4:**

**
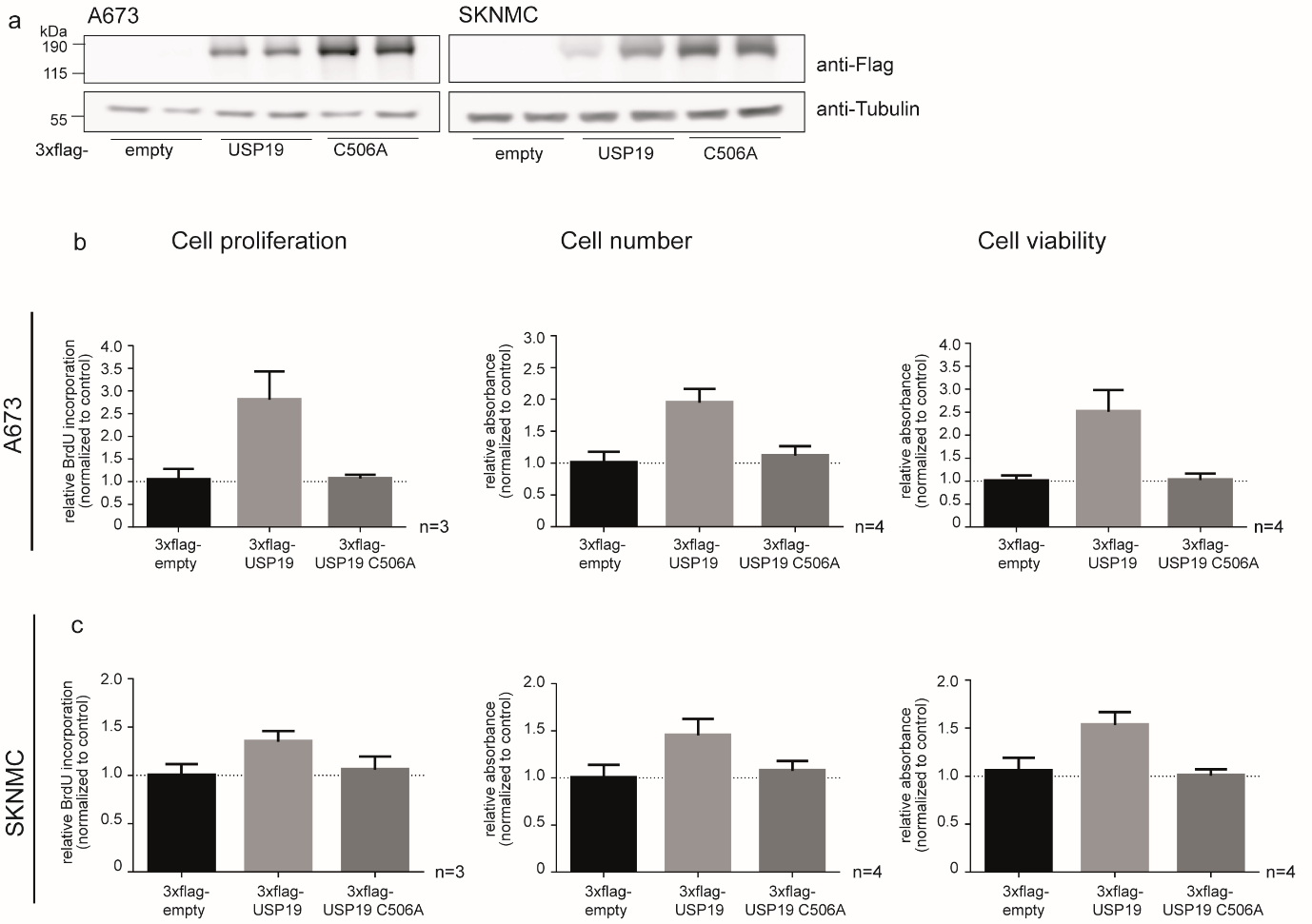
**

**Supplementary Fig. 4:** (a) A673 and SKNMC were transiently transfected with control, 3xflag-USP19 or 3xflag-USP19 C506A mutant. Protein expression was analyzed by western blotting using anti-Flag antibody. (b-c) Cells were analyzed for cell proliferation by BrdU incorporation, for cell number by crystal violet staining and for cell viability by WST1 incubation with n=3 or 4 independent experiments, error bars as SD.

**Supplementary Fig. 5:**


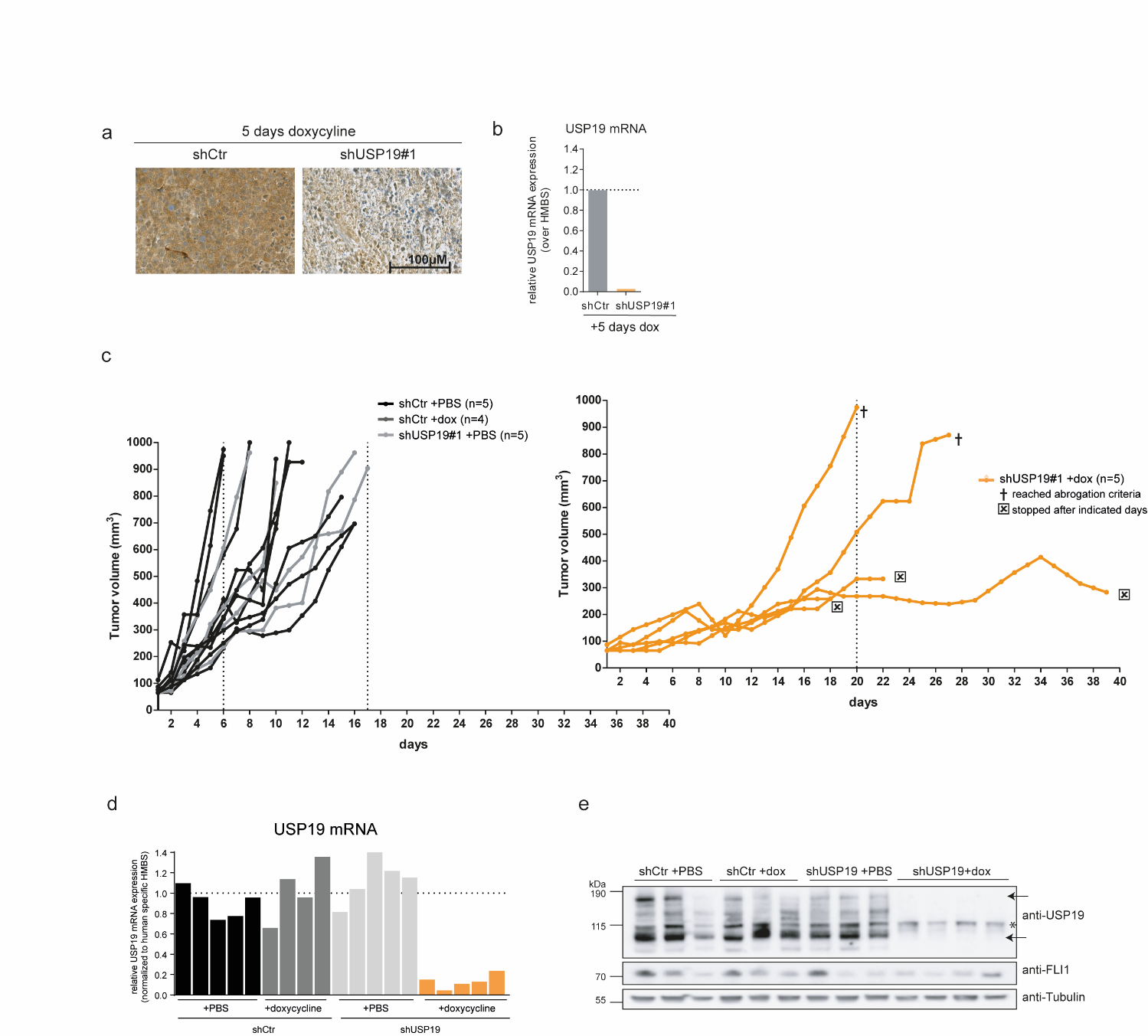


**Supplementary Fig. 5: Depletion of USP19 delays tumor growth *in vivo*.** (a-b) Immunohistochemical analysis and mRNA expression of representative sections of SKNMC shControl or shUSP19#1 tumors treated with doxycyline for five days using an USP19 antibody. (c) Single tumor growth rate of indicated cell lines and treatment of subcutaneously injected SKNMC cells. (d-e) Tumors lysates were analyzed by (d) western blotting with anti-USP19 and anti-FLI1 antibodies, arrows indicate specific bands, asterix marks unspecific band. (e) mRNA expression was determined by quantitative RT-PCR normalized to HMBS.

**
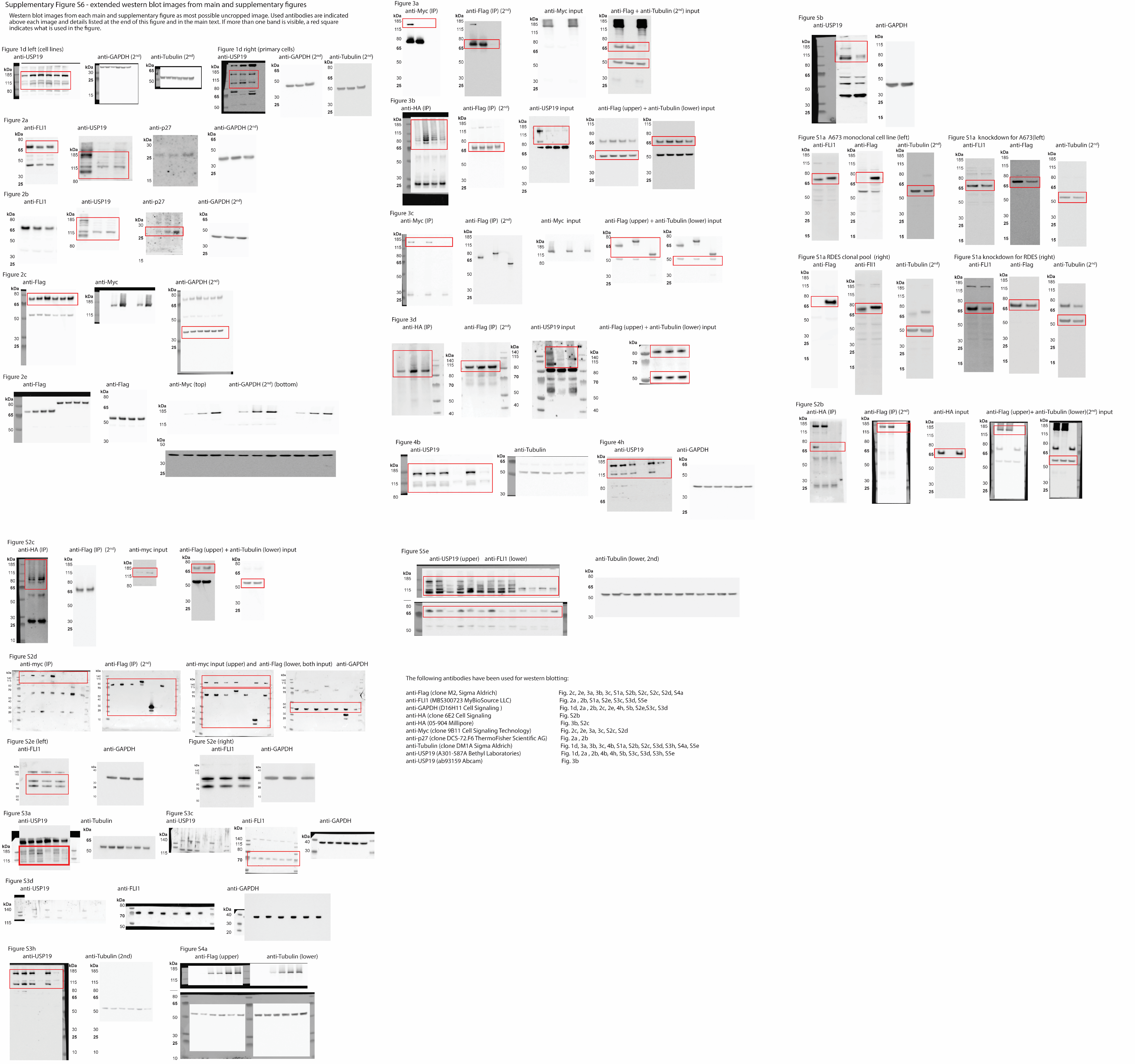
**

**
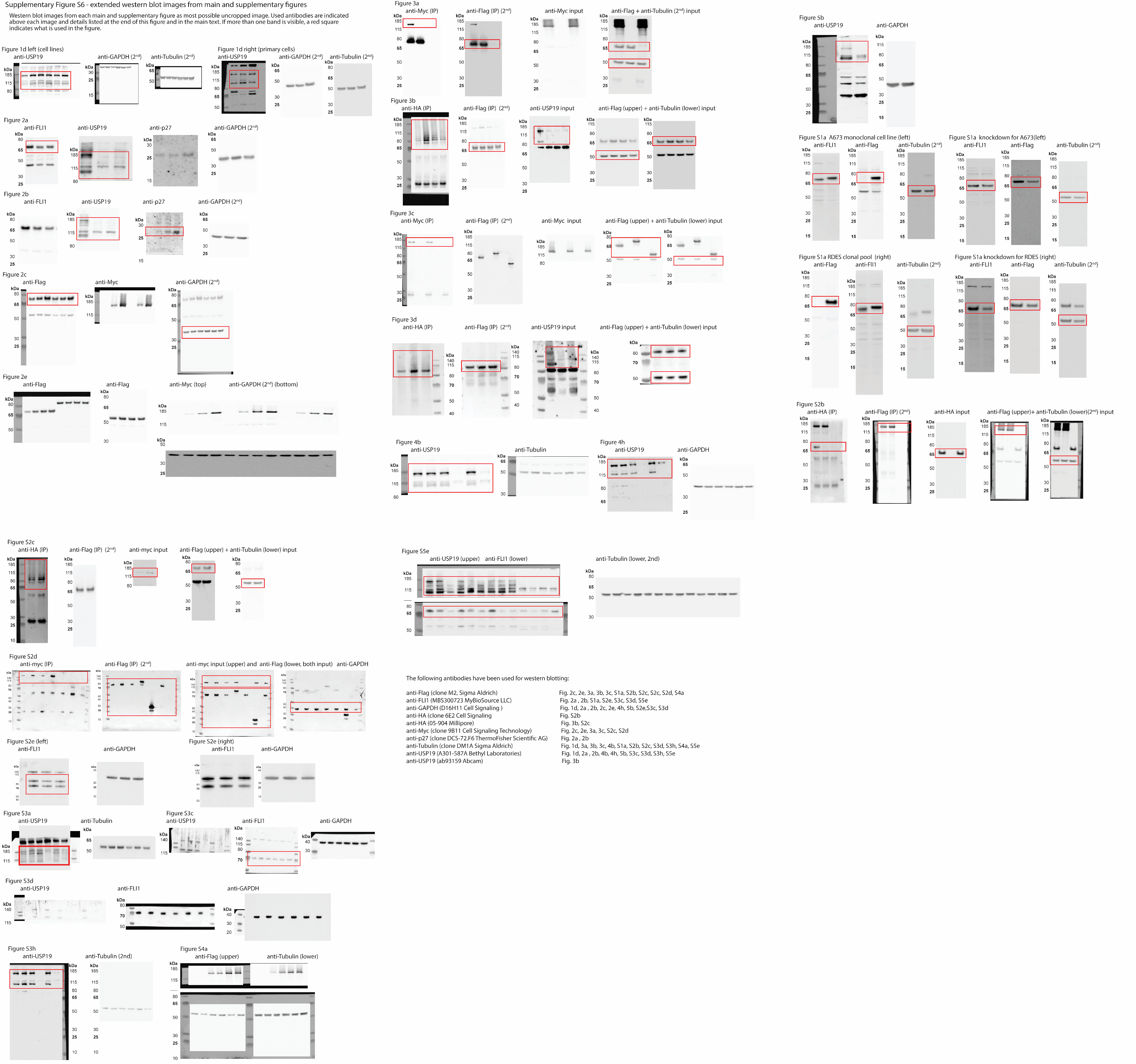
**

**
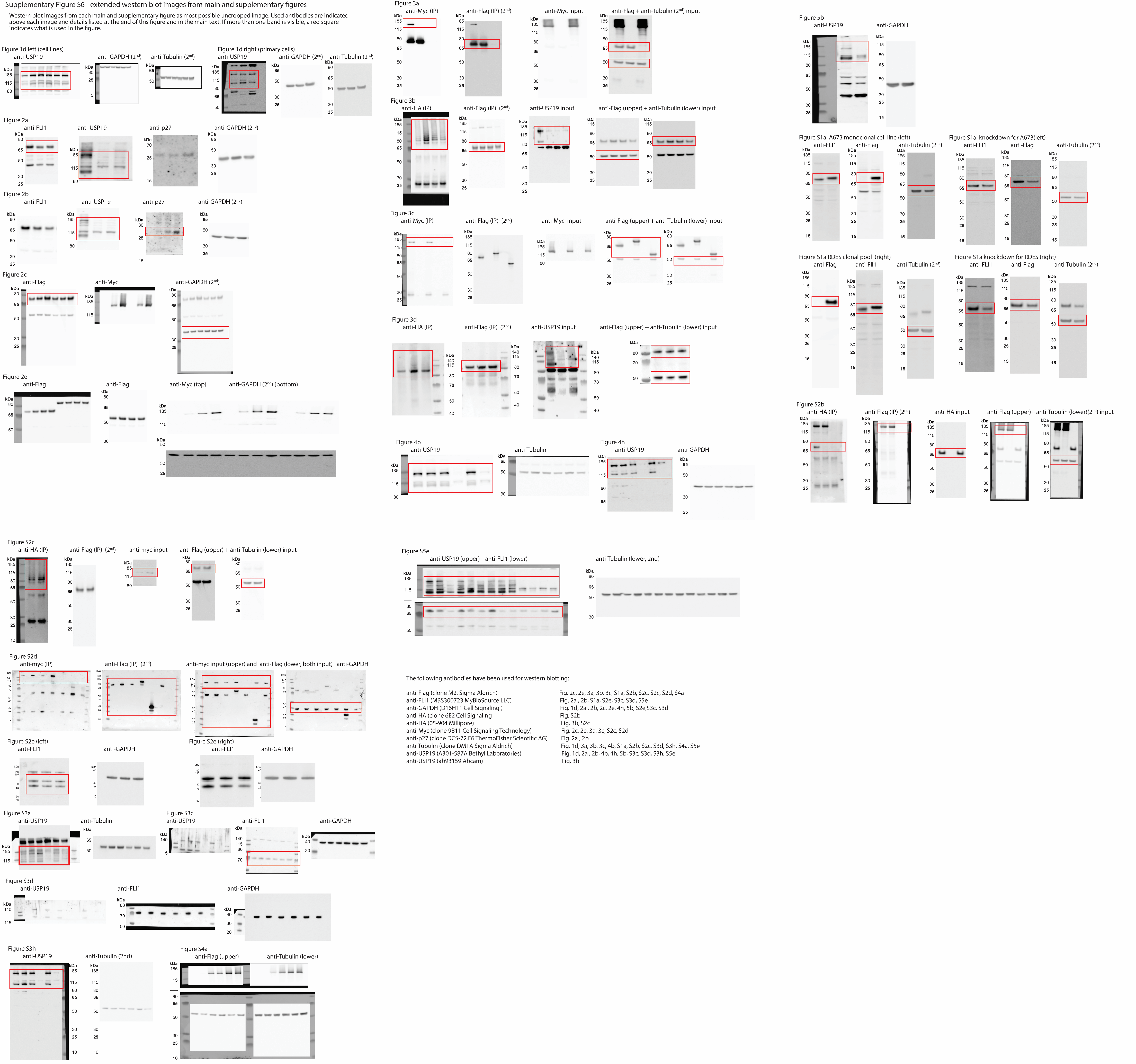
**

**
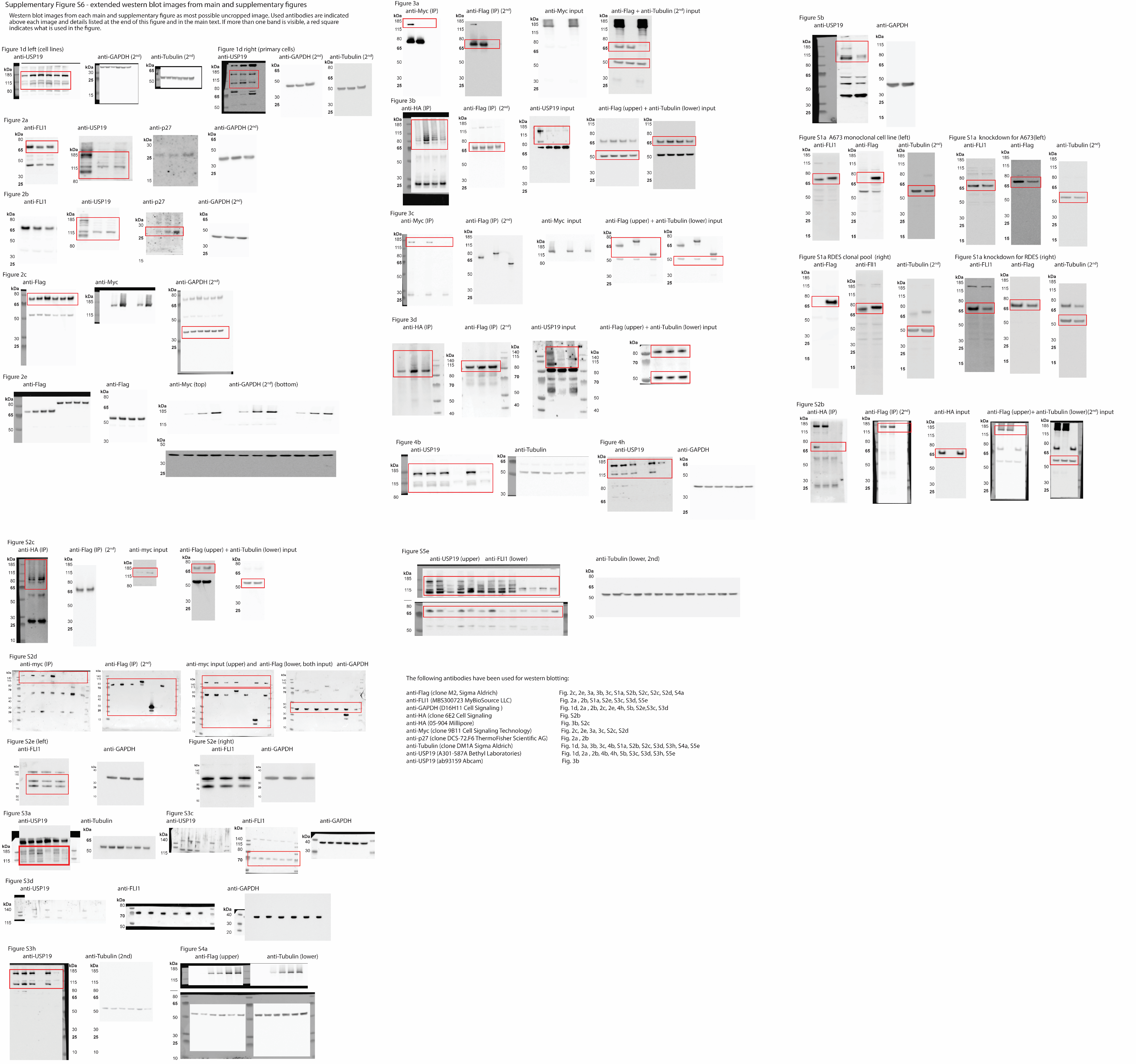
**

**
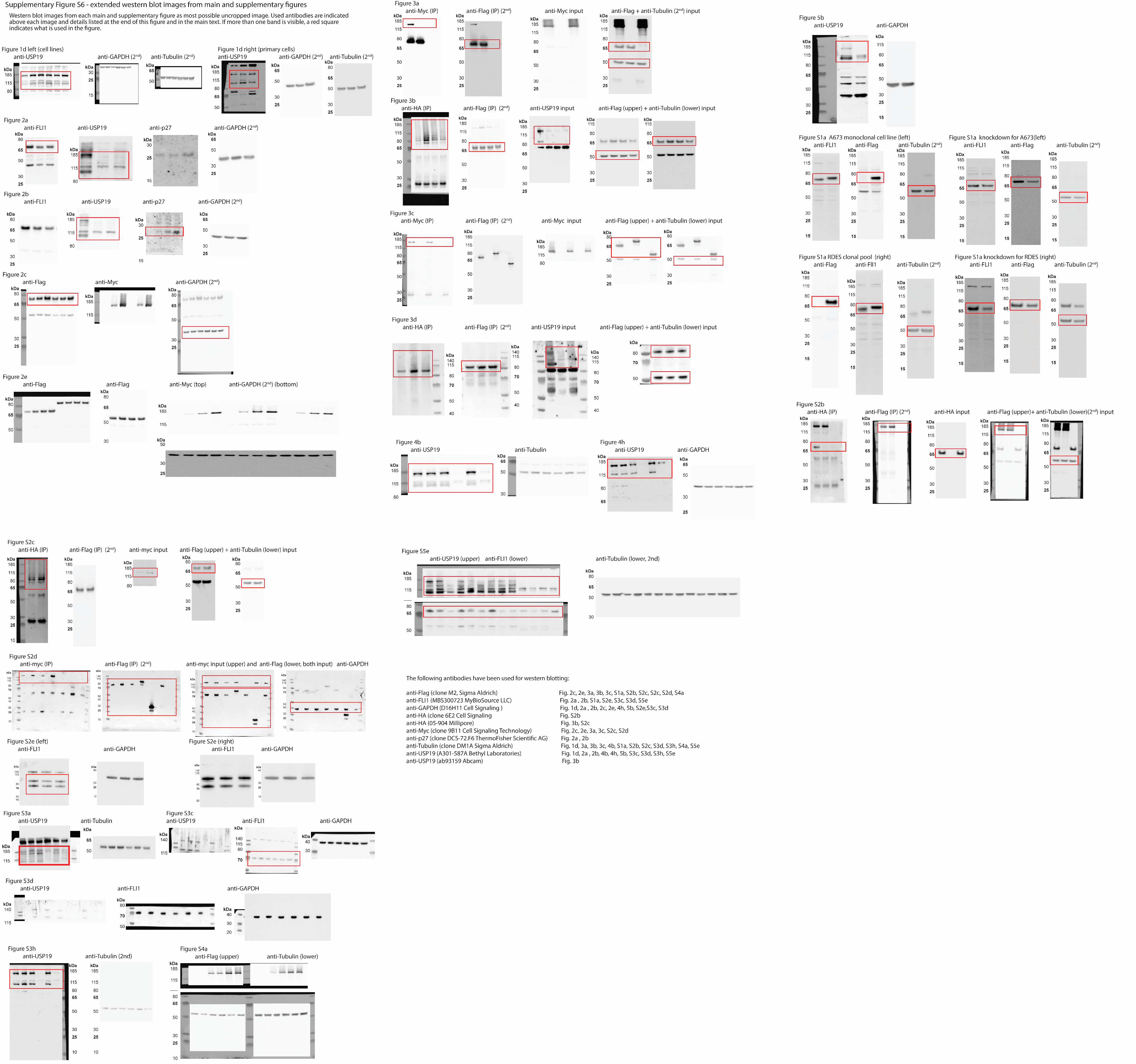
**

**Supplementary Table ST1:** Publicly available gene expression data sets used to select DUB candidates *in silico*.

| **Sample** | **# of files** | **Affymetrix chip** | **GEO accession** | **Authors** |
| --- | --- | --- | --- | --- |
|  |  | | | |
| **Cell lines:** |  |  |  |  |
| SKNMC cells | 1 | Hg-U133A | GSE1824 | Staege et al. 2004 |
| A673 cells (siRNA controls) | 2 | Hg-U133A | GSE7007 | Prieur et al. 2004 |
| A673 cells (luciferase knockdown controls) | 4 | Hg-U133A | GSE4565 | Smith et al. 2006 |
| A673 cells (DMSO controls) | 17 | Hg-U133A | GSE6930 | Stegmaier et al. 2007 |
| EW24, SKNMC cells (shRNA controls) | 4 | Hg-U133A | GSE7007 | Tirode et al. 2007 |
| A673, RD ES cells | 6 | Hg-U133p2 | https://cabig.nci.nih.gov/  caArray_GSKdata/ | Greshock et al. 2010 |
| Different Ewing cell lines | 10 | Hg-U133p2 | GSE17679 | Savola et al. 2011 |
| Different Ewing cell lines | 5 | Hg-U133p2 | GSE36139 | Barretina et al. 2012 |
|  |  |  |  |  |
| **Tumor samples:** |  |  |  |  |
| Tumor samples | 5 | Hg-U133A | GSE1825 | Staege et al. 2004 |
| Tumor samples | 27 | Hg-U133A | GSE7007 | Tirode et al. 2007 |
| Tumor samples | 35 | Hg-U133p2 | GSE12102 | Scotlandi et al. 2009 |
| Tumor samples | 90 | Hg-U133p2 | GSE17679 | Savola et al. 2011 |

**Supplementary Table ST2:** SiRNA library used for screening.

|  |  | **Silencer® Select siRNA:** | | |
| --- | --- | --- | --- | --- |
| **No.** | **Gene name** | **#1** | **#2** | **#3** |
| 1 | USP1: ubiquitin specific peptidase 1 | s14724 | s14725 | s14723 |
| 2 | USP10: ubiquitin specific peptidase 10 | s17367 | s17368 | s17369 |
| 3 | USP11: ubiquitin specific peptidase 11 | s15741 | s15739 | s15740 |
| 4 | USP12: ubiquitin specific peptidase 12 | s47597 | s47595 | s47596 |
| 5 | USP13: ubiquitin specific peptidase 13 | s17130 | s17131 | s17129 |
| 6 | USP14: ubiquitin specific peptidase 14 | s17358 | s17359 | s17360 |
| 7 | USP15: ubiquitin specific peptidase 15 | s19338 | s19339 | s19340 |
| 8 | USP16: ubiquitin specific peptidase 16 | s20808 | s20810 | s20809 |
| 9 | USP19: ubiquitin specific peptidase 19 | s21341 | s21340 | s21339 |
| 10 | USP20: ubiquitin specific peptidase 20 | s21336 | s21337 | s21338 |
| 11 | USP21: ubiquitin specific peptidase 21 | s25689 | s25690 | s223719 |
| 12 | USP22: ubiquitin specific peptidase 22 | s23566 | s23568 | s230744 |
| 13 | USP3: ubiquitin specific peptidase 3 | s19343 | s19341 | s19342 |
| 14 | USP33: ubiquitin specific peptidase 33 | s22873 | s22874 | s22872 |
| 15 | USP4: ubiquitin specific peptidase 4 | s14681 | s14683 | s14682 |
| 16 | USP46: ubiquitin specific peptidase 46 | s35034 | s35035 | s35036 |
| 17 | USP5: ubiquitin specific peptidase 5 | s15595 | s15596 | s15597 |
| 18 | USP6: ubiquitin specific peptidase 6 | s17363 | s17361 | s17362 |
| 19 | USP7: ubiquitin specific peptidase 7 | s15439 | s15440 | s15441 |
| 20 | USP8: Ubiquitin specific peptidase 8 | s17372 | s17370 | s17371 |
| 21 | USP9X: ubiquitin specific peptidase 9, X-linked | s15743 | s15742 | s15744 |
|  |  |  |  |  |
|  |  |  |  |  |
| pos. control | EWS (siRNAs only for region exons 1-7) | s4887 | s4888 |  |
| pos. control | EWS/Fli1 | s5266 | s5268 |  |
| pos. control | EWS/FLI1 breakpoint specific siRNA  (Prieur et al. 2004) | 5′ GGC AGC AGA ACC CUU CUU A-dCdG 3' | | |
|  |  |  |  |  |
| neg. control | Silencer® Select Negative Control No. 1 siRNA |  |  |  |
| neg. control | Silencer® Select Negative Control No. 2 siRNA |  |  |  |
| neg. control | Silencer® Negative Control No. 3 siRNA |  |  |  |
| neg. control | Silencer® Negative Control No. 4 siRNA |  |  |  |
|  |  |  |  |  |
| transf. Control | Silencer® KIF11 (Eg5) siRNA (Human, Mouse, Rat) |  |  |  |
